# Evaluation of a newly developed oral and maxillofacial surgical robotic platform (KD-SR-01) in head and neck surgery: a preclinical trial in porcine models

**DOI:** 10.1038/s41368-024-00318-8

**Published:** 2024-07-10

**Authors:** Zhongkai Ma, Zhiyong Guo, Zhangfan Ding, Chang Cao, Jialu He, Heyi Tang, Yufei Hua, Jiawei Hong, Qiang Shen, Grace Paka Lubamba, Xiaoyi Wang, Zheng Yang, Guiquan Zhu, Chunjie Li

**Affiliations:** 1 State Key Laboratory of Oral Diseases & National Center for Stomatology & National Clinical Research Center for Oral Diseases & Department of Head and Neck Oncology West China Hospital of Stomatology, Sichuan University, Chengdu, China; 2 State Key Laboratory of Oral Diseases & National Center for Stomatology & National Clinical Research Center for Oral Diseases & West China Hospital of Stomatology, Sichuan University, Chengdu, China; 3 School of Mechanical Engineering, Sichuan University, Chengdu, China; 4 Department of Oral and Maxillofacial Surgery, Faculty of Dental Medicine, Hospital of the University of Kinshasa, Kinshasa, Democratic Republic of the Congo

**Keywords:** Preclinical research, Oral cancer detection

## Abstract

Traditional open head and neck surgery often leaves permanent scars, significantly affecting appearance. The emergence of surgical robots has introduced a new era for minimally invasive surgery. However, the complex anatomy of the head and neck region, particularly the oral and maxillofacial areas, combined with the high costs associated with established systems such as the da Vinci, has limited the widespread adoption of surgical robots in this field. Recently, surgical robotic platform in China has developed rapidly, exemplified by the promise shown by the KangDuo Surgical Robot (KD-SR). Although the KD-SR has achieved some results comparable to the da Vinci surgical robot in urology and colorectal surgery, its performance in complex head and neck regions remains untested. This study evaluated the feasibility, effectiveness, and safety of the newly developed KD-SR-01, comparing it with standard endoscopic systems in head and neck procedures on porcine models. We performed parotidectomy, submandibular gland resection, and neck dissection, collected baseline characteristics, perioperative data, and specifically assessed cognitive workload using the NASA-TLX. None of the robotic procedures were converted to endoscopic or open surgery. The results showed no significant difference in operation time between the two groups (*P* = 0.126), better intraoperative bleeding control (*P* = 0.001), and a significant reduction in cognitive workload (*P* < 0.001) in the robotic group. In conclusion, the KD-SR-01 is feasible, effective, and safe for head and neck surgery. Further investigation through well-designed clinical trials with long-term follow-up is necessary to establish the full potential of this emerging robotic platform.

## Introduction

Traditional open head and neck surgeries have long been effective but are associated with a significant disadvantage-noticeable facial scarring, which potentially causes considerable distress for patients in both physical appearance and mental well-being.^1–4^ The emergence of minimally invasive surgery in the 1980s represented a crucial turning point, as it led to the gradual integration of endoscopic technology into clinical practice. For head and neck surgery, endoscopic techniques have greatly improved aesthetic outcomes by altering the location or shortening the length of incisions.^5–7^ However, it is imperative to recognize that endoscope-assisted surgery in this region has its limitations.^8^ Challenges include two-dimensional visualization, which complicates the accurate identification and differentiation of the complex and layered anatomy in the head and neck region. Additionally, the restricted maneuverability of endoscopic instruments poses difficulties in performing delicate operations, such as dissecting tiny facial nerves. Furthermore, the non-ergonomic design of these endoscopic instruments can lead to increased surgeon fatigue. Collectively, these factors have hindered the widespread adoption of endoscopic techniques in head and neck surgeries.

Robot surgery, hailed as the “third technological revolution” in the surgical domain, offers significant advancements in overcoming various limitations of traditional methods.^9^ The integration of a 3D imaging system provides a clearer view of the surgical field, enhancing the differentiation of various tissues.^10^ The master–slave control system, a core component of robotic surgery, minimizes instrument tremor and reduces surgeon fatigue, thereby facilitating smoother and more precise operations.^11^ Currently, almost all robot-assisted head and neck surgeries are performed using surgical robots like the da Vinci system, which, although originally developed for urological and abdominal procedures, stands out as the most successful in this field.^12–15^ Unlike the larger and more regular cavities in the abdominal areas, which are well-suited for robotic and endoscopic procedures facilitated by carbon dioxide insufflation, the compact space of the head and neck area poses distinct challenges. While the da Vinci robot, approved for head and neck surgeries in 2005, has achieved success in procedures involving the pharynx,^16^ larynx,^17^ and thyroid,^18,19^ its adoption in other head and neck surgeries is still limited. This region is densely packed with numerous tissues, all of which are intricately intertwined.^20^ Conducting minimally invasive surgeries here often necessitates the creation of channels by surgeons, adding complexity to the use of robotic systems and requiring a greater degree of flexibility in surgical instruments.^21^ Moreover, this region contains delicate blood vessels and nerves, demanding even higher precision in surgical instrument handling. However, the availability of the da Vinci system is restricted in many hospitals, particularly in developing regions, owing to its high equipment and maintenance costs.

**Fig. 1:**
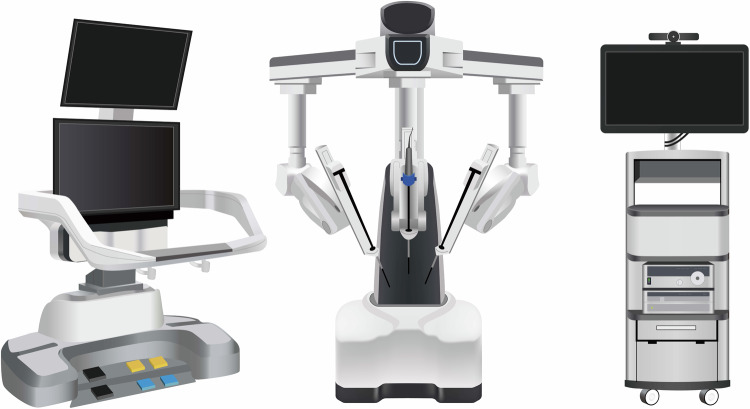
KD‐SR‐01 robotic surgical system. The KD-SR-01 robotic system composes of a surgical console integrating two master manipulators and a 3D high-definition monitor (left), a patient card with three robotic arms (middle), and an imaging system (right)

The KangDuo Surgical Robot-01 (KD-SR-01), a newly developed robotic platform from China, is a master–slave system consisting of an open surgeon console, a three-arm robotic operation cart, and a 3D video imaging system (Fig. 1). The surgeon console is equipped with two screens: the lower one displays real-time intraoperative 3D images. The upper one can connect to external devices, providing access to preoperative images or 3D reconstruction models based on preoperative CT scans. This setup offers valuable assistance during surgeries. Additionally, the open console design facilitates optimal hand–eye coordination and enables surgeons to avoid maintaining a fixed posture, thereby preventing neck fatigue.^22^ The surgical instruments mounted on the robotic arms feature seven degrees of motion, enabling them to execute actions that surpass the capabilities of human hands. Furthermore, the development and production costs for KD-SR-01 are estimated to be only 25%–30% of those for the da Vinci,^23^ making it a more accessible option for budget-conscious medical facilities. Currently, the KD-SR systems have demonstrated promising application prospects across some surgical procedures. In the field of urology, KD-SR-assisted radical prostatectomy showed no biochemical recurrence over an 11-month follow-up period, and the surgical success rates for pyeloplasty and ureterovesical reimplantation were 96% and 92%, respectively, over follow-up periods of 29.5 and 11.5 months.^24^ In the field of abdominal, the efficacy and safety of KD-SR-01 for colon cancer have been proven to be on par with the da Vinci robotic system.^25^ However, given the complexities of the head and neck region, the feasibility and safety of the KD-SR remain unproven.

In this study, we utilized the KD-SR-01 robotic surgical platform to perform parotidectomy, submandibular gland (SMG) resection, and selective neck resection on porcine models. By comparing these operations with standard endoscopic surgery, we sought to rigorously evaluate the safety, effectiveness, and operation efficiency of the KD-SR-01. This pioneering preclinical investigation not only tests the viability of this newly developed robotic surgical platform but also seeks to illuminate its potential to revolutionize the field of head and neck surgery, paving the way for future clinical adoption.

## Results

The KD-SR-01 was employed in the robotic group, and each robotic operation was executed in accordance with the procedure outlined below:

1. Body position: all pigs were positioned in the lateral decubitus position for each operation (Fig. 2a).

**Fig. 2:**
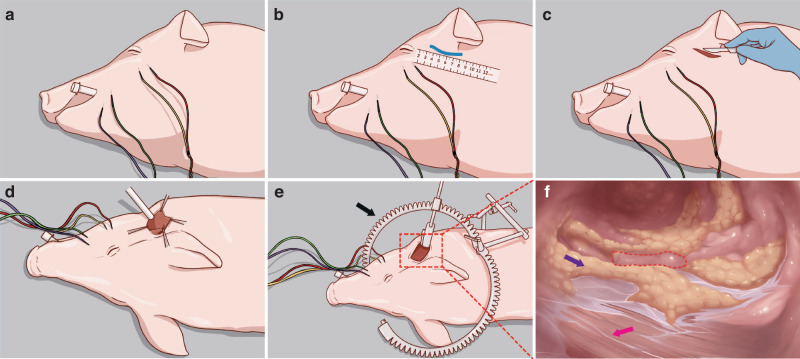
Surgical preparation for robotic group. **a** Placement of the positive terminals and searching units of the IFNM to assess the muscles innervated by the facial nerve. **b** An arch incision about 5 cm long was made along with the ear root and 4–6 cm away from the posterior margin of the ramus mandibulae. **c**, **d** Using traditional surgical instruments to incise the skin and create the surgical cavity. **e** Installing a specially designed surgical retractor to support the cavity allowed easier access for surgical instruments. **f** Constructing a sufficiently large cavity to achieve adequate exposure of the posterior border of the parotid gland and the brachiocephalic muscle. Black arrow: special surgical retractor developed by Zhu. Pink arrow: brachiocephalic muscle. Purple arrow: adipose tissues. Red dotted line: parotid gland
2. Intraoperative facial nerve monitoring (IFNM): placement of the positive terminals and searching units of the IFNM (Fig. 2a).
3. Incision and cavity building: due to the limited natural space in the head and neck region, it was necessary to construct the cavity for the operation before commencing with the robotic and endoscopic procedures. An arch incision was made along the ear root, 4–6 cm away from the posterior margin of the mandibular ramus (Fig. 2b). Using surgical instruments to open up layers of tissue to get sufficient operating space (Fig. 2c, d). A specialized surgical retractor (5Rmed, Chengdu) was used to lift and secure the skin flap, facilitating the completion of cavity formation (Fig. 2e). The posterior boundary of the parotid gland was exposed after the completion of cavity construction (Fig. 2f).
4. Docking: the surgical robot was positioned properly with laser guidance. When the robot reaches the appropriate position, the surgical instruments are installed in turn. After completing the above steps, the surgeon can start the operations (Fig. 3).

**Fig. 3:**
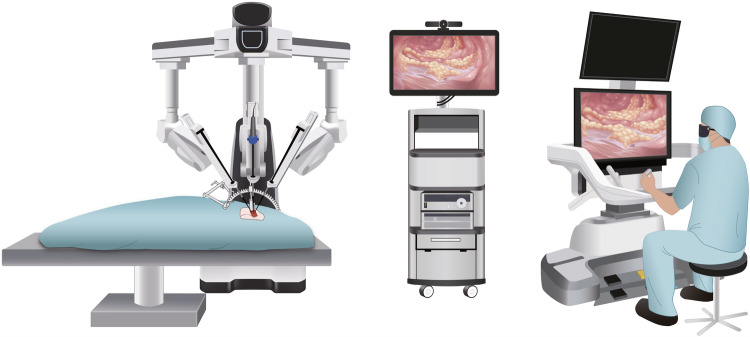
Schematic diagram of head and neck surgery using the KD-SR-01 robotic system. An open surgical console enables surgeons to precisely and synchronously control the surgical arms and instruments with passive polarizing glasses without flexion of the neck. Patient card (left); imaging system (middle); surgical console (right)
5. Parotidectomy (Video 1): Lifting the posterior boundary of the parotid gland, we carefully proceeded to detach and separate it toward the inferoposterior lower boundary (Fig. 4a). During this process, the parotid lymph node was exposed and meticulously dissected (Fig. 4b). Deep to the parotid gland lay the superficial jugular vein (Fig. 4c). We carefully separated the deep lobe of the parotid gland from the vein and surrounding connective tissues. The robotic instruments, designed to bend at greater angles, facilitate the clamping and dissection of the anterior boundary of the gland. Under the enhanced visualization of the robotic system, tiny blood vessels become clearly visible and can be easily managed with energy instruments (Fig. 4d, e). We continued the dissection upward to the upper boundary of the parotid gland, and the facial nerve branch was located on the deep surface of the parotid gland (Fig. 4d, e). The parotidectomy was completed with the removal of the parotid gland (Fig. 4f).

**Fig. 4:**
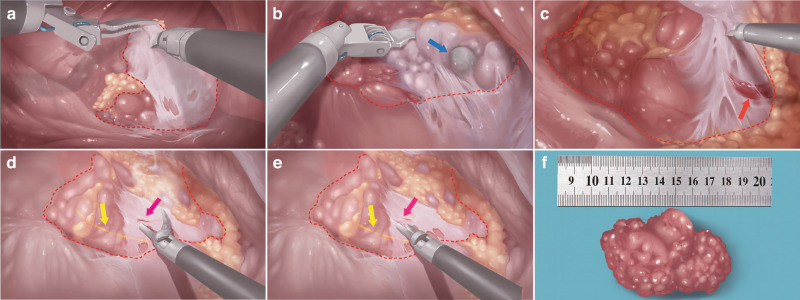
Surgical technical of parotidectomy with the robotic system in a porcine model. **a** Lifting the posterior pole of the parotid gland. **b** During the dissection of the gland’s inferior boundary, the parotid lymph node was exposed. **c** Separating the deep lope of the parotid gland from the superficial jugular vein and other connective tissues. **d** During robot-assisted parotidectomy, tiny vessels can be clearly identified. e. Given the maneuverability provided by the surgical robot, the instrument can easily clamp small blood vessels in the clear surgical field. **f** Parotid gland specimen. Blue arrow: parotid lymph node. Red arrow: superficial jugular vein. Pink arrow: tiny blood vessel. Yellow arrow: facial nerve branch. Red dotted line: parotid gland
6. SMG resection (Video 2): after completing the parotidectomy, the dissection continued anteriorly and inferiorly, revealing the dense capsule of the SMG. The marginal mandibular branch of the facial nerve can be seen around the SMG. Carefully dissect to preserve the integrity of the nerve. Unlike in humans, where blood vessels are commonly found surrounding the SMG, the porcine anatomy lacks such distinct structures around the gland. This anatomical difference allowed for more straightforward separation and complete resection of the SMG from all directions with the help of multi-freedom robotic instruments. The submaxillary lymph node is exposed during the dissection of the anterior boundary of the SMG. We continued to dissect the bottom boundary of the SMG, completed the resection, and removed the gland.
7. Selective neck dissection (Video 3): after SMG resection, we continued with the dissection downward to perform the neck dissection. Given the anatomical differences between pig and human necks, we opted for selective neck dissection rather than attempting extensive dissection. Initially, we established the posterior and upper boundaries of the selective neck dissection based on the position of the deep anterior edge of the cervical cutaneous muscle and then performed the dissection. Dissect the tissue along the omohyoid muscle. During the dissection of the upper boundary, the lingual vein adhering to the neck dissection tissue was identified. This vessel was carefully separated from the tissue using energy instruments. After separating the neck dissection tissue from the underlying mylohyoid muscle, the neck dissection tissue was removed. Then, the IFNM was used to confirm that the nerve remained intact. When we placed the metal probe near the nerve, a signal was generated on the monitor screen, and an alarm sounded (Fig. S1a, b). In contrast, if the metal probe came into contact with non-nerve tissue, the IFNM did not generate any signals or alarms (Fig. S1c, d).
8. Seaming: the incision was closed without the aid of the surgical robot.

In this study, all 12 surgical procedures were completed successfully and none of the surgeries in the robotic group required conversion to endoscopic or open procedures, and similarly, none of the surgeries in the endoscopic group necessitated conversion to open surgery.

**Fig. 5:**
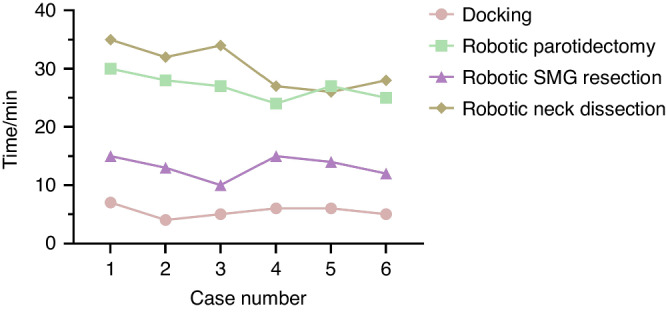
Trend of time of different robotic procedures using the KD-SR-01 system

As detailed in Table 1, a total of six Bama miniature pigs were used in this study. Their average age was 18.00 months, with a mean weight of 43.33 kg. The mean baseline heart rate and preoperative oxygen saturation were 58.67 and 96.50%, respectively. About the perioperative outcomes (Table 2), the mean length of incision was (4.62 ± 0.23) cm in the robotic group and (4.65 ± 0.24) cm in the endoscopic group (*P* = 0.765). Regarding the time required for cavity construction, no significant difference was found between the two groups (12.17 ± 0.75) min vs. (11.67 ± 1.21) min, *P* = 0.414). The docking time in the robotic group was (5.50 ± 1.05) min. There were no significant differences in the time of parotidectomy, SMG resection, and neck dissection. Although the mean total operation time in the robotic group was 6.67 min longer than that in the endoscopic group, there was no statistically significant difference between the two groups (88.00 ± 5.76) min vs. (81.33 ± 7.81) min, *P* = 0.126). The total estimated blood loss was (2.00 ± 0.71) mL in the robotic group and (4.67 ± 1.17) mL in the endoscopic group (*P* = 0.001). As shown in Fig. 5, the average duration of robotic neck dissection and parotidectomy decreased from 33.7 and 28.3 min for the first three cases to 27 and 25.3 min for the last three cases, respectively. This trend is expected to continue with improved coordination.

**Table 1:** Baseline characteristics of all porcine models

Variables	Total porcine models (*n* = 6)
Age, months (SD)	18.00 (0.32)
Sex	
Male	1 (17%)
Female	5 (83%)
Weight/kg (SD)	43.33 (1.03)
Baseline heart rate, beats per min (SD)	58.67 (1.97)
Preoperative oxygen saturation/% (SD)	96.50 (1.05)

*SD* standard deviation

**Table 2:** Perioperative outcomes between robotic and endoscopic groups

Variables	Robotic group	Endoscopic group	*P* value
Length of incision/cm (SD)	4.62 (0.23)	4.65 (0.24)	0.765
Time of operative procedures/min (SD)			
Cavity construction	12.17 (0.75)	11.67 (1.21)	0.414
Docking	5.50 (1.05)	–	–
Parotidectomy	26.83 (2.14)	27.83 (2.79)	0.502
SMG resection	13.17 (1.94)	13.00 (1.67)	0.877
Neck dissection	30.33 (3.83)	28.33 (4.67)	0.557
Suture	7.67 (0.82)	8.17 (0.75)	0.296
Total operation time	88.00 (5.76)	81.33 (7.81)	0.126
EBL during different surgeries/mL (SD)			
EBL during parotidectomy	0.75 (0.27)	1.92 (0.49)	0.001
EBL during SMG resection	0.42 (0.50)	0.75 (0.52)	0.283
EBL during neck dissection	0.83 (0.26)	2.00 (0.71)	0.008
Total EBL	2.00 (0.71)	4.67 (1.17)	0.001
Operative heart rate, beats per min (SD)	48.00 (0.89)	47.33 (1.03)	0.260
Operative oxygen saturation/% (SD)	95.67 (0.82)	95.17 (0.75)	0.296
Facial nerve damage	None	None	–

*SD* standard deviation, *SMG* submandibular gland, *EBL* estimated blood loss

In this study, the KD-SR-01 was assessed for its ability to protect the facial nerve throughout the whole operation process. During robotic operations, the facial nerve was identified and safeguarded with the assistance of the imaging system (Table 2). In terms of workload, the National Aeronautics and Space Administration-Task Load Index (NASA-TLX) workload assessment demonstrated that the surgeon experienced significantly more stress in the endoscopic group than in the robotic group, in nearly all aspects except for temporal demand (Table 3). Furthermore, as indicated in Table 4, the NASA-TLX scores of the assistant for robotic head and neck surgery were notably lower than those for endoscopic surgery across all six perspectives (*P* < 0.05).

**Table 3:** The surgeon’s NASA-TLX cognitive workload assessment between robotic and endoscopic surgeries

Variables	Robotic group (*n* = 6)	Endoscopic group (*n* = 6)	*P* value
NASA-TLX score, median (range)			
Mental demand	4 (4, 5)	6 (4, 7)	0.045
Physical demand	4 (3, 5)	7 (6, 8)	0.001
Temporal demand	4 (3, 5)	4 (3, 5)	0.695
Performance	3 (3, 4)	6 (5, 7)	0.003
Effort	5 (4, 5)	7 (6, 7)	0.003
Frustration	4 (4, 5)	7 (6, 7)	<0.001
Total scores	24.5 (23, 27)	35.5 (34, 38)	<0.001

**Table 4:** The assistant’s NASA-TLX cognitive workload assessment between robotic group and endoscopic group

Variables	Robotic group (*n* = 6)	Endoscopic group (*n* = 6)	*P* value
NASA-TLX score, median (range)			
Mental demand	2 (1, 2)	4 (4, 5)	<0.001
Physical demand	1 (1, 2)	7 (6, 8)	<0.001
Temporal demand	2 (2, 3)	4 (3, 5)	0.001
Performance	1 (1, 3.)	4 (3, 5)	0.006
Effort	2 (1, 2)	6 (5, 7)	<0.001
Frustration	2 (1, 2)	4 (4, 5)	<0.001
Total scores	10.5 (9, 11)	29.5 (28, 31)	<0.001

## Discussion

In our study, we conducted different surgeries on both sides of the same pig. This was done to ensure that the technical difficulty of the operations was comparable between the robotic and endoscopic groups.

The primary outcome was the total operative time, and we found no significant or clinically meaningful difference between the two groups. In fact, the total operative time in the robotic group was only ~6 min longer than that in the endoscopic group. Our results were in line with those reported by Dai et al., who conducted a study involving 12 porcine models to compare KD-SR-01 robotic partial nephrectomy and laparoscopic partial nephrectomy.^26^ In their study, although the operative time in the robotic group was 11 min longer than that in the laparoscopic group, they also found no significant difference between the two groups. Notably, when we removed the docking time in our study, the time required for surgical procedures was almost identical between the robotic and endoscopic groups. As demonstrated by the above findings, operative times were not necessarily prolonged when using this newly developed surgical robotic platform in porcine models. However, in real-world clinical settings, it remains uncertain whether robotic surgery can match the surgical duration of conventional procedures.^27–29^ Kim et al. posited that extensive training can markedly decrease the duration of robotic surgeries. Their research demonstrated significant reductions in the average operation time associated with robot-assisted neck dissections as experience accumulated.^30^ One study even revealed that, with extensive training of the robot, surgical duration could be reduced by up to 40%.^31^

Robotic surgery comes highly recommended not only for its efficiency but, more importantly, for its potential to enhance aesthetic outcomes, improve bleeding control, and minimize complications, among other benefits.^32,33^ Given the extensive blood flow in the head and neck region, effective bleeding control and maintaining a clear surgical field are crucial for robotic surgery. Lim et al. observed notably lower estimated blood loss using the da Vinci system, especially in complex cases like post-styloid parapharyngeal space tumors.^29^ Although it has not been used in human head and neck surgery, the KD-SR-01 has achieved bleeding control in colonic surgeries compared to that of the da Vinci robot.^25^ Our results demonstrated that this system was also well in controlling bleeding in head and neck surgery in the porcine model. This enhancement in blood loss control can be attributed to the sophisticated capabilities of robotic systems, which encompass features such as tremor reduction, 3D imaging, and surgical instruments with a wider range of motion. These attributes collectively empower surgeons to more accurately identify and dissect blood vessels within the correct anatomical planes. However, it is worth noting that in our study, during SMG resection, we observed no statistically significant differences in estimated blood loss between the two groups. This can be attributed to the distinct anatomical characteristics of the porcine SMG, which possesses a well-defined boundary with the surrounding tissue and relatively fewer blood vessels in proximity. Besides bleeding control, the protection of critical nerves is also very important for some head and neck surgeries.^7,34,35^ IFNM was utilized and the results demonstrated that the facial nerve was protected well during operations.

In this study, all surgical incisions made behind the ear root and mandibular ramus in porcine models were designed to simulate the trans-hairline approach used in human surgery. This specific incision technique was initially proposed by Woo for their endoscope-assisted procedures, demonstrating its feasibility.^21^ Subsequently, Yang reported on a series of 24 patients who underwent robotic sialoadenectomy using the trans-hairline approach, highlighting its practical application in robotic head and neck surgery.^36^ Then, Yang et al. conducted studies that demonstrated the surgical configuration and procedures of robotic trans-hairline SMG resection using flexible, single-port, and multi-arm systems.^37^ Although the trans-hairline incision cannot achieve a completely scarless body surface, it is virtually undetectable when concealed within the hair. Moreover, this type of incision is associated with fewer complications and effectively balances aesthetic and therapeutic outcomes.^38^ According to our study, we have successfully completed all operations using the simulated trans-hairline approach in porcine models with the KD-SR-01. We intend to continue using this approach in the forthcoming clinical trial, which was registered at www.chictr.org.cn (ChiCTR2300076776).

In terms of cognitive workload, the surgical robot demonstrates significant advantages over standard endoscopic surgery. The robotic systems provide surgeons with a more comfortable operating posture, unlike standard endoscopy, which often requires maintaining awkward and physically demanding positions for extended periods. By allowing surgeons to sit at a console with optimal ergonomic support, robotic systems reduce physical strain and fatigue. Additionally, robotic arms offer a greater range of motion than human hands, and the instruments can articulate far beyond the capabilities of standard endoscopic tools. This enhanced maneuverability allows for more precise dissections, reducing the cognitive workload associated with the fine control of instruments. Furthermore, robotic systems include mechanisms that filter out hand tremors from the surgeon’s movements, enhancing the precision of surgical tasks. This steadiness is particularly valuable during delicate procedures and can significantly reduce the mental effort required to achieve precise movements. For assistants, robotic surgery alleviates the need to hold instruments in a fixed position for long durations, a common requirement in standard endoscopic surgery. As a result, the surgical team can concentrate more on the strategic aspects of the operation rather than the technical challenges, potentially leading to improved decision-making and outcomes.

It is important to note that these experimental findings do not directly establish that the robot can produce similar results in humans, primarily due to the anatomic disparities between humans and pigs. First, while the parotid gland of the pig is larger, it lacks the precise blood vessels and main facial nerve running through it. In humans, the parotid gland contains many blood vessels and features a more intricate facial nerve structure, which heightens the complexity of the surgery. Second, the SMG in pigs features a denser envelope and presents a well-defined boundary with the surrounding blood vessels. Third, the larger lower jaw in pigs, in comparison to that of adult humans, results in a greater distance for the surgical instruments to traverse from behind the pig’s ear to the leading edge of the parotid gland and the lower boundary of the neck. This extended distance also contributes to the heightened surgical complexity. These dissimilarities underscore the necessity of conducting robotic clinical trials to bridge the gap between animal models and human applications.

In the realm of surgical robots, comparisons between the newly developed surgical robotic system and the da Vinci system are inevitable. Regarding postoperative outcomes, Li et al. conducted a comparative study on robot-assisted partial nephrectomy (RAPN), a common and representative procedure, to assess these two systems.^39^ Their study, comprising 99 RAPN surgeries (49 with KD-SR and 50 with da Vinci), indicated that the KD-SR achieved efficacy comparable to the da Vinci robot, with no significant differences in complication rates between the groups. Additionally, Fan’s study on robot-assisted radical prostatectomy (RARP) using the KD-SR also reported similar short-term oncological and functional outcomes, albeit with a longer operation duration compared to the da Vinci system.^40^ They suggested that the operational efficiency of the KD-SR might be enhanced through more comprehensive training, as evidenced by their extensive experience gained from over 400 RARP cases with the da Vinci system. In addition to its successful application in urological surgeries, the KD-SR-01 is as effective as the da Vinci system for colon cancer procedures.^25^ The performance of the KD-SR-01 in these procedures therefore raises expectations for its application in head and neck surgeries.

This study had some limitations that need to be considered. First, the extrapolation of our results from preclinical models to human application may be limited due to inter-species differences.^41^ As such, the necessity for further clinical trials is evident, and we are actively working to promote such trials to bridge the gap between preclinical and clinical settings. Second, in our effort to assess the potential applicability of the KD-SR-01 in head and neck surgery, we conducted three distinct procedures. This approach, while informative, resulted in relatively small sample sizes for each procedure. Lastly, when evaluating the KD-SR-01, it is worth noting that comparing it with established platforms like the da Vince system may provide a more objective assessment.

This newly developed robotic system has preliminarily demonstrated its technical feasibility, safety, and validity in head and neck surgery. This innovative system offers distinct advantages from an ergonomic perspective, particularly when compared to traditional endoscopic surgery. Despite the promising potential of KD-SR-01, it is imperative to underscore the necessity for a meticulously designed clinical trial, inclusive of an extensive follow-up period. This would be pivotal in rigorously evaluating the true efficacy and utility of the KD-SR-01. Conducting such a comprehensive study is essential to gain a holistic understanding of the system’s strengths and limitations within a clinical setting.

## Methods

### Experimental animals, study design, and ethics

In this animal study, six healthy pigs were utilized. Surgical interventions were conducted on one side using endoscopic techniques and on the other side using surgical robots. A lottery method was adopted for the randomization process. One container was assigned to determine the initial side to operate on (right or left), while another container was designated to decide the surgical method.

All surgical procedures were performed by an experienced head and neck oncology surgeon with extensive experience in endoscopic head and neck surgery (over 200 cases) and more than 200 h of training on the KD-SR system. Before this experiment, the surgeon completed five parotidectomies, five SMG resections, and five selective neck dissections using KD-SR-01 to acquaint himself with the machine and pig anatomy. Two veterinarians were responsible for the perioperative management of experimental animals.

During the study, the first operation was initiated at 9:00 a.m. daily. After each operation (comprising one parotidectomy, one SMG resection, and one selective neck dissection), the surgeon and the assistant had a 2-h rest period to minimize the impact of previous surgeries on subsequent ones. Up to two operations were conducted per day.

This animal study was authorized by the Experimental Animal Center and approved by the Ethics Committee of West China Hospital of Stomatology, Sichuan University (No. WCHSIRB-D-2023-053).

### Procedures

Parotidectomy, SMG resection, and selective neck dissection were performed sequentially in each group. We collected a comprehensive set of variables: baseline characteristics (age, weight, sex, baseline heart rate, preoperative oxygen saturation), operative data (length of incision, time of cavity building, docking time, parotidectomy time, SMG resection time, neck dissection time, suture time, total operation time, estimated blood loss, operative heart rate, operative oxygen saturation, facial nerve injury), and cognitive workload assessment.

The docking time was defined as the duration from the initial movement of the robotic cart to the successful docking of the last cannula with its corresponding robotic arm. The cavity building time started with an incision of skin and ended with a retractor installed. The IFNM was employed to detect whether facial nerve injury occurred during operations. In the absence of nerve damage, an electrical impulse was generated when the probe touched the facial nerve. Estimated blood loss was measured by the suction liquid and soaked gauze dressing. All the animals were allowed for food intake 6 h after surgery. The NASA-TLX was used to assess the mental workload of the surgeon and the assistant after each operation. This tool is widely utilized to measure the perceived workload of individuals performing specific tasks and encompasses six key dimensions. Mental demand refers to the cognitive effort required, including thinking, decision-making, calculating, and remembering. Physical demand encompasses the physical effort required, such as moving, pushing, and controlling. Temporal demand captures the pressure from time constraints, reflecting the level of urgency, and rush an individual feels. Performance is the individual’s perception of their success in achieving self-set or externally set goals, considering the outcome, and required effort. Effort denotes the degree of hard work the individual feels they have put in to achieve their level of performance. Frustration measures the level of stress, annoyance, or dissatisfaction experienced during the task. This assessment was conducted through a subjective questionnaire.

### Outcomes

In this study, the primary outcome assessed was the total operation time, comprising the time spent on cavity construction, docking, parotidectomy, SMG resection, neck dissection, and suturing. The secondary outcomes encompassed (1) estimated blood loss, (2) the occurrence of facial nerve injury, and (3) the mental workload experienced by both the surgeon and the assistant.

### Statistical analysis

Statistical analyses were carried out by the SPSS Statistics software (version 26.0; IBM Corp, Armonk, NY, USA). Continuous variables were reported as either the mean with standard deviation (SD) or the median with minimum and maximum values, contingent on the normal distribution of the variables. To ascertain the significance of differences, statistical tests including the Wilcoxon rank-sum test and paired or unpaired *t*-tests were employed. A *P* value < 0.05 was considered statistically significant.

### Supplementary information

Supplemental Information

Video 1: Robot-assisted parotidectomy

Video 2: Robot-assisted submandibular gland resection

Video 3:Robot-assisted neck dissection
