# Supplementary material for: Evaluation of a newly developed oral and maxillofacial surgical robotic platform (KD-SR-01) in head and neck surgery: a preclinical trial in porcine models
Source: Int J Oral Sci. 2024 Jul 10;16:51. doi: 10.1038/s41368-024-00318-8 (PMC11237157; doi:10.1038/s41368-024-00318-8)
Supplement: Supplementary file 1 — Supplemental Information [file 41368_2024_318_MOESM1_ESM.docx]

Supplementary information:

**Evaluation of a Newly-developed Oral and Maxillofacial Surgical Robotic Platform (KD-SR-01) in Head and Neck Surgery: A Preclinical Trial in Porcine Models**

**Running title:** Newly-developed surgical robot in head and neck surgery.

Zhongkai Ma^1,#^, Zhiyong Guo^1,#^, Zhangfan Ding^1^, Chang Cao^1^, Jialu He^1^, Heyi Tang^1^, Yufei Hua^1^, Jiawei Hong^2^, Qiang Shen^2^, Grace Paka Lubamba^1,4^, Xiaoyi Wang^1^, Zheng Yang^2^, Guiquan Zhu^1,*^,Chunjie Li^1,*^

^1^ State Key Laboratory of Oral Diseases & National Center for Stomatology & National Clinical Research Center for Oral Diseases & Department of Head and Neck Oncology West China Hospital of Stomatology, Sichuan University, Chengdu, Sichuan, China

^2^ State Key Laboratory of Oral Diseases & National Center for Stomatology & National Clinical Research Center for Oral Diseases & West China Hospital of Stomatology, Sichuan University, Chengdu, Sichuan, China

^3^ School of Mechanical Engineering, Sichuan University, Chengdu, Sichuan, China

^4^ Department of Oral and Maxillofacial Surgery, Faculty of Dental Medicine, Hospital of the University of Kinshasa, Kinshasa, Democratic Republic of the Congo

^#^ These authors contributed equally: Zhongkai Ma and Zhiyong Guo

^*^ These authors are co-corresponding authors.


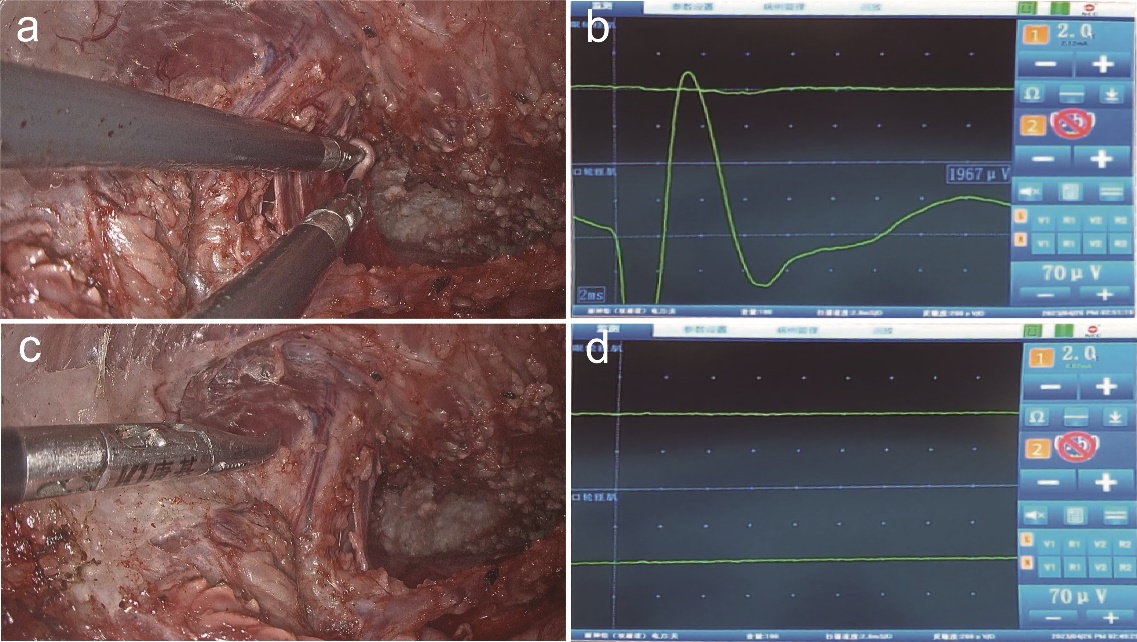


**Figure S1**. Using the IFNM to detect nerves. a. Placing the metal probe near the nerve. b, Signal displayed on the nerve monitor indicate that the nerve is intact. c. Placing the metal probe near the non-nerve tissue. d, No signal was detected on the nerve monitor.
